# Location Privacy-Preserving Scheme in IoBT Networks Using Deception-Based Techniques

**DOI:** 10.3390/s23063142

**Published:** 2023-03-15

**Authors:** Basmh Alkanjr, Imad Mahgoub

**Affiliations:** Department of Electrical Engineering & Computer Science, Florida Atlantic University, 777 Glades Road, Boca Raton, FL 33431, USA

**Keywords:** location privacy, IoBT, security, dummy ID, dummy location, pseudonym

## Abstract

The Internet of Battlefield Things (IoBT) refers to interconnected battlefield equipment/sources for synchronized automated decision making. Due to difficulties unique to the battlefield, such as a lack of infrastructure, the heterogeneity of equipment, and attacks, IoBT networks differ significantly from regular IoT networks. In war scenarios, real-time location information gathering is critical for combat effectiveness and is dependent on network connectivity and information sharing in the presence of an enemy. To maintain connectivity and guarantee the safety of soldiers/equipment, location information must be exchanged. The location, identification, and trajectory of soldiers/devices are all contained in these messages. A malicious attacker may utilize this information to build a complete trajectory of a target node and track it. This paper proposes a location privacy-preserving scheme in IoBT networks using deception-based techniques. Dummy identifier (DID), sensitive areas location privacy enhancement, and silence period concepts are used to minimize the attacker’s ability to track a target node. In addition, to consider the security of the location information, another security layer is proposed, which generates a pseudonym location for the source node to use instead of its real location when sending messages in the network. We develop a Matlab simulation to evaluate our scheme in terms of average anonymity and probability of linkability of the source node. The results show that the proposed method improves the anonymity of the source node. It reduces the attacker’s ability to link the old DID of the source node with its new DID. Finally, the results show further privacy enhancement by applying the sensitive area concept, which is important for IoBT networks.

## 1. Introduction

The Internet of Battlefield Things (IoBT) is a newly emerging field that uses Internet of Things (IoT) technologies for defense purposes [1]. IoT technology allows devices to collaborate to monitor physical or environmental conditions [2]. In addition, it allows sensing and transmitting information, enabling real-time decision making in military operations [3].

Due to the highly heterogeneous nature of the IoBT environment in terms of devices, network protocols, platforms, connectivity, and other factors, there are difficulties associated with trust, security, and privacy [3]. Since communication infrastructure might be unavailable, these entities communicate with one another utilizing device-to-device (D2D) communications to send and receive sensitive information, including location information [4].

The requested location information queries may be vulnerable to attacks, and the adversaries can use the location information to follow users in myriad ways or reveal their personal information to third parties. Securing the location of IoBT entities is essential to protect military equipment from enemy ambushes; if this information is leaked, it could result in the failure of an important mission or even the loss of life. Additionally, a well-secured location helps the military to create an efficient attack and defense strategy, leading to successful mission outcomes. Because of these security precautions, cybersecurity is now crucial for protecting IoBT equipment from assaults that could impact military operations. Many methods have been proposed to protect the identity of the node and its location. The use of security deception in cyber defense is a promising strategy that has attracted the interest of researchers [5]. Some of the deception methods include, but are not limited to. pseudonyms ID, dummy location, and silence period.

Pseudonym ID methods are frequently used in a variety of security research [6] to address the identity and location privacy issues of the user [7]. In this technique, each node has pseudonym identities that it uses to communicate with other nodes instead of its actual identity [8]. The node regularly changes its pseudonym for every new communication. As a result, the attacker cannot identify who exactly its target is [8]. Therefore, it is anticipated that the method will be able to offer untraceability, privacy, and user anonymity to its user [6]. In this manner, the node data and information are protected from access by unauthorized parties [9].

However, due to the adversary’s ability to monitor and link the nodes that are sending their locations even after they change their pseudonyms, utilizing pseudonyms will unfortunately not completely solve all privacy issues. As a solution, a silence period is proposed to protect the identity information of the node once the pseudonym ID is about to expire. It is a time period during which the node does not participate in any activity. During the silence period, the node will change its dummy ID with a new one. Thus, the silence period prevents the attacker’s linkability to link the old pseudonym with the new one [8].

A dummy location in another method is proposed to protect the real location information. Dummy location is a strategy that intends to deceive the adversary with fictitious locations [10]. By sending a dummy location instead of the real one to the service provider, the attacker will not be able to distinguish the real one from the dummy one. Therefore, this scheme is able to provide anonymity and location privacy to the user.

To the best of our knowledge, there is no existing work that addresses the identity and location privacy issues in IoBT networks. In this paper, we propose a scheme that uses dummy ID, sensitive areas location privacy enhancement, and silence period concepts to enhance location information and identity privacy for IoBT entities. The sensitive area concept further enhances the location privacy in battlefield areas that are sensitive.

In this paper, we make the following contributions:

- We develop a scheme to protect the node’s identity by using dummy ID, silence period, and sensitive areas location privacy enhancement concepts.
- We generate a pseudonym location for each node in the IoBT environment to protect the node’s real location information.
- We introduce a new metric, average probability of linkability per DID change of a source node, to measure how successful the attacker is in linking the source node with its new DID after the silence period.
- To evaluate our scheme, we use average anonymity and average probability of linkability per DID change of a source node.
- We develop a Matlab simulation to validate our proposed scheme.

The rest of our work is organized as follows. Section 2 presents an overview of the related work. Section 3 illustrates the proposed method. The proposed method analysis, including the performance metrics, simulation environment, parameters, network assumption, security analysis, and simulation results and analysis, are included in Section 4. Finally, Section 5 concludes the paper.

## 2. Related Work

In this section, we provide an overview of location privacy-preserving mechanisms. Many methods have been proposed to protect location information as explained below:

Instead of using actual vehicle IDs, vehicles can share essential information while maintaining their privacy by using pseudonyms [11]. To maintain location privacy, the work in [12] employed a strong pseudonym change method that guarantees unlinkability. The suggested solution confuses the attacker during the pseudonym updating phase. It combines the principles of “hiding inside the crowd” and “location obfuscation.” The authors in [13] offered a novel comprehensive pseudonym changing scheme that takes advantage of the vehicle context and pattern of the current traffic to determine the best scenario for switching pseudonyms. A dynamic pseudonym swap zone (DPSZ)-based location privacy-preserving technique was suggested in [14] in which each vehicle can generate a temporary pseudonym swap zone using DPSZ on demand to exchange pseudonyms with any other vehicle within a certain zone. Ref. [15] proposed using a genetic algorithm to create a set of pseudonyms using the crossover approach. The set is created for vehicles at different time intervals by crossing an initial pair of pseudonyms (GA). Ref. [16] proposed a novel scheme for centralized pseudonym changing for location privacy in vehicle-to-anything communication. In [17], the authors discussed changing the identity of the vehicle with a mix zones-based authentication protocol for location privacy. In [18], a concerted silence-based location privacy preserving scheme for Internet of Vehicles (CSLPPS) was proposed to guarantee pseudonyms and prevent the adversary from tracking the participation node in IoV. The method is based on entering a silent period to change the identifiers of cooperative vehicles at the same time.

The researchers in [19] used a cryptographic technique to present a reliable and effective scheme for preserving location privacy. Because the identity of the user is hidden from the location-based service (LBS) provider and fog server, it can protect the user’s private information. Both AES with one-time-pad keys and IBE were used in communication to ensure the confidentiality and integrity of both requesting and receiving data. Ref. [20] suggested a (P 2 FHE-AES) method for LBS inquiry called privacy-preserving fully homomorphic encryption over advanced encryption standards for the purpose of encouraging drivers to utilize this service without worrying about being tracked. To avoid location privacy leakage from sensory data, the authors in [21] designed an encrypted data recovery scheme based on homomorphic encryption as part of their work. Several works used cryptography/encryption as a solution to protect the location information such as [22,23,24,25].

Ref. [26] proposed a scheme called dummy location provider (DLP) that consists of three algorithms: spread, shift, and switch. Spread and shift are responsible for creating deceptive dummies and trajectories, while switch replaces users’ actual locations with dummy trajectories before submitting them to the LBS. The authors in [27] proposed query-based dual location privacy in vehicle ad hoc networks (VANETs). They used the circle-based dummy generation (CBDG) algorithm to create some dummy locations before sending the query to a trusted third party. The local differential privacy technique is used in [28] to present a novel privacy-aware framework for aggregating indoor location data. In this technique, user location data are altered locally in the user’s device and then transferred to the aggregator. As a result, neither a server nor any potential attackers are aware of the user’s geolocation. Ref. [29] suggested a blockchain privacy protection crowdsourcing solution that can secure employees’ location privacy and increase job completion rates. In addition to using blockchain technology’s anonymous capabilities to hide users’ identities, this system creates private blockchains to distribute members’ transaction records and selects jobs across several private blockchains to prevent the deletion of members’ transaction information. The authors in [30] used blockchain technology for the Internet of Vehicles (IoV) to protect the task and worker’s location privacy. The proposed system not only protects the privacy of worker locations but also improves task completion success rates. The work in [31] offered a new approach to protecting location privacy for mobile crowdsourcing systems that enhanced privacy protection and service quality. Other works used blockchain for location privacy purposes, such as [32,33,34,35,36].

Both works in [12,37] employed crowd-blending and obfuscation strategies to maintain location anonymity in VANETs. Ref. [38] proposed a coordinate transformation-based scheme. Location privacy was implemented using the CBDG algorithm and a trusted third party. The proposed method takes advantage of both obfuscation and anonymity methods by using a two-step authentication procedure to share location data among neighboring vehicles. A mobile device conducts some basic geometric functions (shifting, rotation) before relaying its positions to the LBS provider. Refs. [3,39,40,41,42] are other efforts that use obfuscation mechanisms for location privacy.

Table 1 illustrates the comparison of our work to recent deception-based related works where “√” means the method is used, “×” means the method is not used. As we can see, all the existing schemes presented in the table address location privacy issues in IoT, WSN, and IoV, and none address location information and identity privacy of entities in IoBT networks. Additionally, no exiting works consider securing the location information if an entity enters a sensitive area, which can be critical, particularly in IoBT environments. In this paper, we propose a scheme that uses dummy ID, sensitive area, and silence period concepts to enhance location information and identity privacy for IoBT entities.

## 3. Overview of the Proposed Method

### 3.1. Network Assumptions

The network assumptions of our work are:

1. The nodes use D2D communication to communicate with the gateways.
2. The gateways are mobile.
3. The communication between the gateways is secure.
4. The gateways know the real identities of each other.
5. The gateways are powerful devices and have controls on the nodes that are located in their communication range.
6. The registration table is distributed and secure, and only authenticated users can access it.
7. The network space is divided into n grid cells. The grid cells are numbered from 2 to n + 1. The gateway nodes know the cells’ locations and numbers.

### 3.2. The Network Architecture:

**Figure 1:**
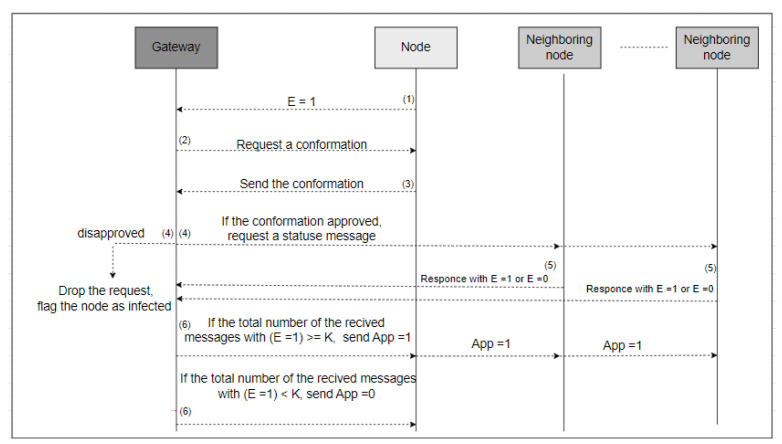
The network architecture steps.

This paper discusses how to protect identity and location information from unauthorized entities to provide a more secure system in the IoBT environment. Dummy ID, sensitive areas location privacy enhancement, and silence period concepts are used to minimize linkability to protect the node identity. Additionally, an alternative location for each node will be generated and used instead of the real location information. The proposed network architecture to request changing the Dummy ID (DID) is shown in Figure 1. Table 2 shows the notations of the proposed work.

### 3.3. The Proposed Method

The proposed method aims to protect both the identity and the location information of each node on the battlefield. First, the network is divided into grid cells, and each grid cell has a unique integer identifier n. The gateways have the same grid cell number as an identifier if they are located in that grid cell when they communicate for the first time with the node for registration purposes. The reason for using this identifier n is that it will be used to generate a pseudonym location, which will be explained later.

**Figure 2:**
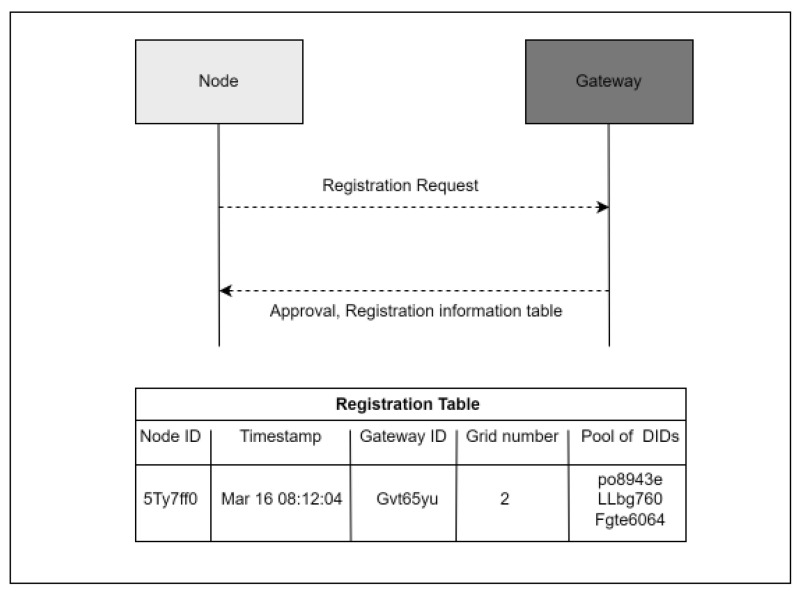
The registration process.

**Figure 3:**
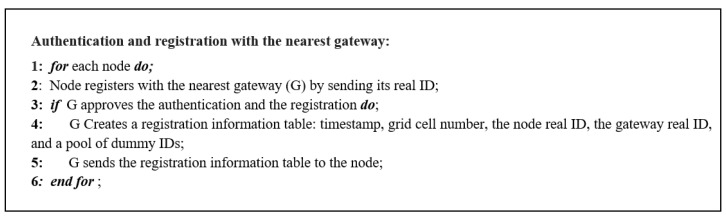
Authentication and registration with the nearest gateway algorithm.

Figure 2 demonstrates the second step; each node is initially required to authenticate itself and register with the nearest gateway G by sending its real ID. Once the registration is approved, the registration information table for each node will be created. The registration information contains the timestamp, the grid cell number, the ID for the node, the ID for the gateway, and a pool of dummy IDs. Then, the gateway will transmit the registration table to the node. Figure 3 shows the algorithm for the authentication and registration step.

**Figure 4:**
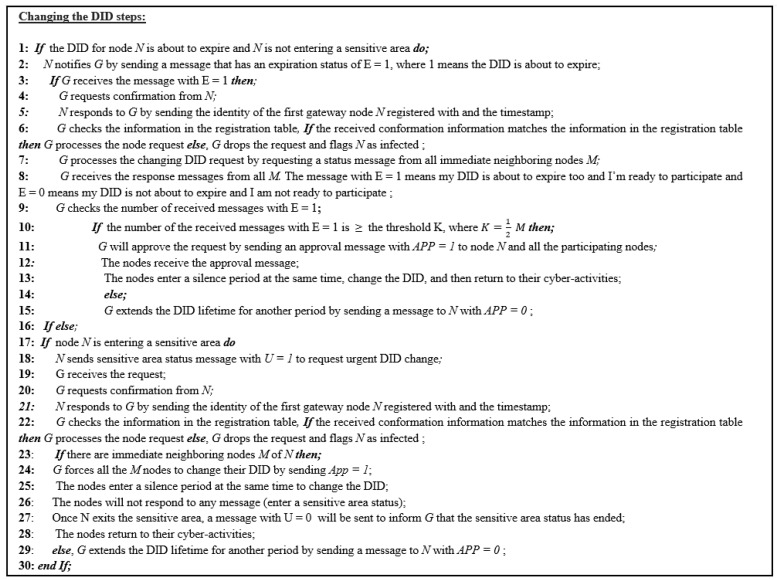
The algorithm for changing the DID.

The DIDs will be used for any communication in the field in place of the real IDs. By using DIDs, the real identity will be protected. However, if the node changes its temporary DID during its cyber-activity, the information could be linked to track the target node. Thus, we propose using a silence period concept to protect the identity information while changing the temporary DID. The changing of the DID must occur under two circumstances as explained below. Figure 4 illustrates the formal algorithm for changing the DID.

- The lifetime of the node DID is about to expire, and node N is not entering a sensitive area.
- The node is about to enter a sensitive area. In this case, the node sends a sensitive area status (U = 1) to change the DID whether its lifetime is about to expire or not. This case will be explained in more detail later.

### 3.4. Silence Period

**Figure 5:**
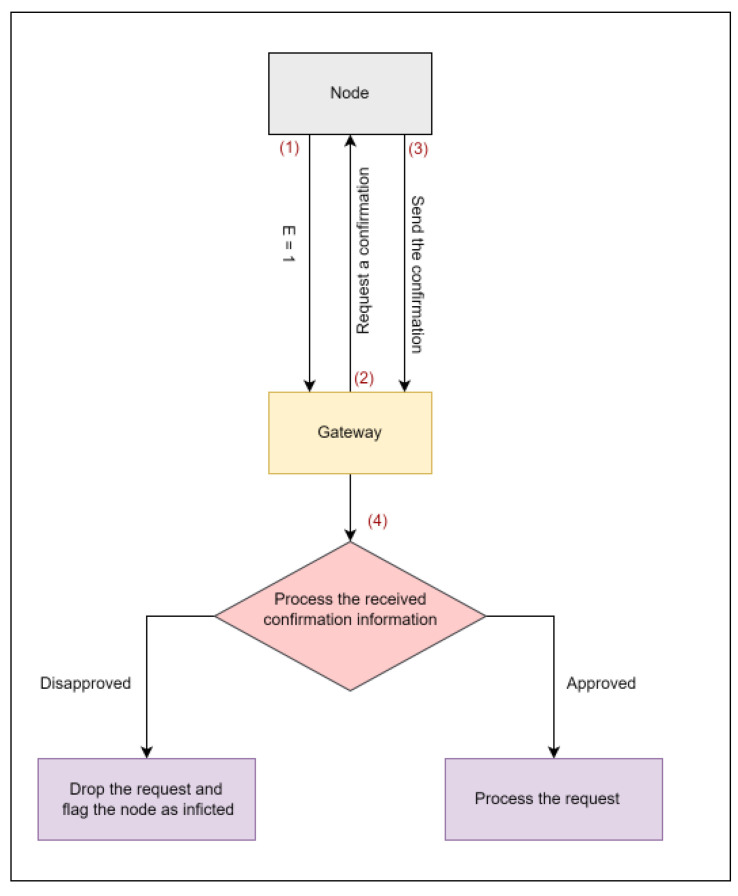
Sending and receiving the confirmation steps.

**Figure 6:**
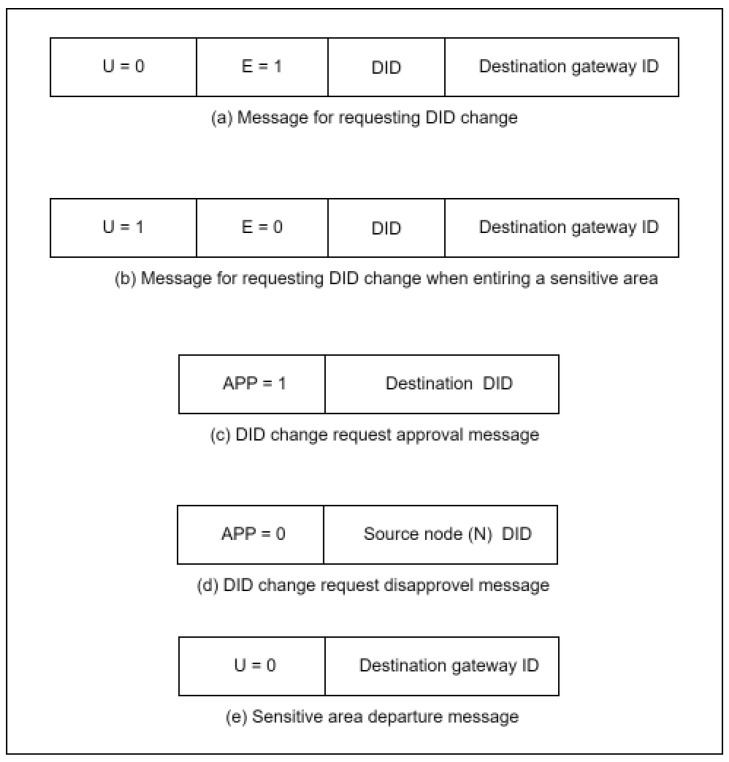
DID change message exchange between nodes and gateways.

The silence period is a method used to protect the identity information of the node once the temporary DID is about to expire. It is a time period during which the node does not participate in any activity. During the silence period, the node will change its DID to prevent linkability. Once the DID is about to expire, the source node, N, must notify the gateway by sending a message that has an expiration status (E = 1). A message with E = 1 means the DID is about to expire, and the node needs approval to change it. Once the gateway receives the message with E = 1, it will request a confirmation from node N, which is the identity of the first gateway the node registered with and the timestamp. Thus, if the confirmation information matches the information in the registration table, the gateway will process the node request. Otherwise, the request will be dropped, and the gateway will flag the node as infected. Figure 5 explains the confirmation process, and Figure 6 illustrates the DID change message exchange between nodes and gateway.

To process the request, the gateway will request a status message from all the *M* immediate neighboring nodes of node N (the first node that requested to change its DID). All M nodes must participate in this task by responding either with E = 1, which means my DID is about to expire too and I am ready to participate, or with E = 0, which means not ready to participate. If the number of the received status messages with E = 1 equals or exceeds the threshold *K*, then the gateway will approve the changing request by sending an approval message with APP = 1 to the source node and the nodes with the same status E = 1. The threshold is defined as in Equation (1):

(1) $$K=\frac{1}{2}\left(M\right)$$

For a source node request to be approved in the non-sensitive area case, it has to have at least least two immediate neighboring nodes out of which one agrees to participate regardless of the total number of nodes in the network.

Once the approval is received, the cooperating nodes and node N will enter a silence period synchronously to change the DID, and then they will return to cyber-activity again. If the number of nodes in the network T is small and there are no enough cooperating nodes, the gateway will extend the DID lifetime for node N for one period (60 s) and send a message to the node with APP = 0. The silence period is used to enhance the identity privacy of the node and reduce the chance of tracking.

### 3.5. Generating Pseudonym Location

**Figure 7:**
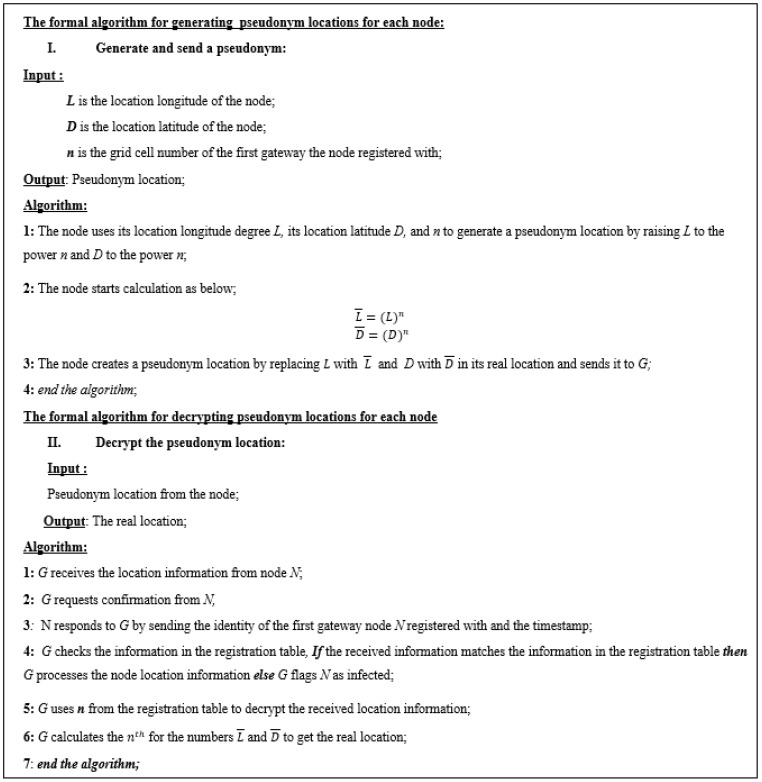
The formal algorithm to generate pseudonym location.

To consider the privacy of the location information, another security layer is proposed in this paper, as shown in Figure 7. Consider a node with longitude *L* and latitude *D*. Assume that n is the grid cell number of the first gateway the node registered with. To generate a pseudonym location for this node, we propose changing *L* and *D* to $\bar{L}$ and $\bar{D}$, respectively, as shown in Equations (2) and (3).

(2) $$\bar{L}={\left(L\right)}^{n}$$

(3) $$\bar{D}={\left(D\right)}^{n}$$

Once the gateway receives the location information from the node, the gateway will ask the node for confirmation as we explained above. The confirmation information is the identity of the first gateway the node registered with and the timestamp. If the confirmation is approved, G will check the registration table and get the grid cell number to decrypt the location information. Using the grid cell number n, the gateway will find (n)th root of $\bar{L}$ and $\bar{D}$ to obtain *L* and *D*.

### 3.6. Sensitive Areas Location Privacy Enhancement

The sensitive area status is also used to secure the location of the node. In this case, once the node enters a sensitive area where the location information of this node must be highly secured, the node has to send an urgent sensitive area status (U = 1). The urgent sensitive area status is a message from node N to change the DID, even if its DID lifetime is not about to expire to inform the gateway that the sensitive area status has started. Once the gateway receives the message and approves the confirmation as mentioned above, G will force all the neighboring nodes to change their DID by sending an approval message (APP = 1). Next, node N and all of its neighboring nodes will enter a silence period status, change their DIDs, and not respond to any messages until they exit the sensitive area. Once node N exits the sensitive area, a message with U = 0 will be sent to the gateway. All the nodes will return to their cyber-activities with the new DIDs. If there are no cooperating nodes, the gateway will extend the DID lifetime for node N and send a message to the node with APP = 0.

## 4. Proposed Method Analysis

### 4.1. Performance Metrics

We propose and use linkability of the source node to measure how successful the attacker is in tracking the source node. In addition, we use average anonymity of the source node metrics to analyze the privacy degree of the location and identity information and to assess the effectiveness of our proposed work:

1. Average anonymity of the source node per DID change (Average AS).
  The Average AS per DID change is defined to be the ratio of the total number of participating nodes in the DID changes to the number of changes.
2. The probability of linkability (PLA).
  We define PLA to be the probability that the attacker will successfully link the source node with its new DID after the silence period.
3. Average probability of linkability (Average PLA).
  We define Average PLA as the ratio of the total values of PLA for all the changes to the number of changes.

### 4.2. Mathematical Model

1. Average AS:
  AS is measured by the number of participating nodes in a DID change.
  AS=NPN
  Based on the above formula, anonymity increases by increasing the number of cooperating nodes. The more nodes that enter the silence period (cooperate nodes) and change their DID with the source node, the lower the probability that the attacker will successfully link the source node DID with its old one.
  The Average AS metric measures the anonymity of the source node N per DID change.
  In the IoBT environment, it is important to increase the anonymity of the source to protect and secure sensitive information. Thus, we propose to measure Average AS in different cases based on the sensitivity of the node’s area. The first case is when the source node enters a non-sensitive area, the second case is when the source node enters a sensitive area where it is important to increase the anonymity of the source node. This feature further enhances the anonymity of the source node. The mathematical models for both cases are as follows:
  - Average AS for non-sensitive area (Average ${\mathrm{AS}}_{\mathrm{NS}}$):
    Here, we derive a mathematical expression for Average ${\mathrm{AS}}_{\mathrm{NS}}$. Let ***i*** donate the DID change number, ${\mathrm{NPN}}_{i}$ denote the number of participating nodes for change ***i***, ***j*** the total number of changes, and ${\mathrm{AS}}_{i}$ be the anonymity of the source node for DID change ***i***.
    $${\mathrm{AS}}_{i}={\mathrm{NPN}}_{i}$$
    In addition, total AS for all the DID changes ***j*** is:
    $$\mathrm{AS}\;\mathrm{total}=\sum_{i=1}^{j}\left({\mathrm{NPN}}_{i}\right)$$
    Therefore, Average AS for a non-sensitive area (Average ${\mathrm{AS}}_{\mathrm{NS}}$) is given by Equation (4) below:
    $$\mathrm{Average}\;{\mathrm{AS}}_{\mathrm{NS}}=\frac{\mathrm{AS}\;\mathrm{total}}{\mathrm{Total}\;\mathrm{number}\;\mathrm{of}\;\mathrm{changes}}$$
    (4) $$\mathrm{Average}\;{\mathrm{AS}}_{\mathrm{NS}}=\frac{1}{j}\sum_{i=1}^{j}\left({\mathrm{NPN}}_{i}\right)$$
  - Average AS for sensitive area (Average ${\mathrm{AS}}_{\mathrm{NS}}$):
    For the sensitive area case, all immediate neighboring nodes ($M_{i}$) of the source node are forced to participate, so
    $${\mathrm{NPN}}_{i}=M_{i}$$
    Thus, Average AS for sensitive area (Average ${\mathrm{AS}}_{S}$) is given by Equation (5) below:
    (5) $$\mathrm{Average}\;{\mathrm{AS}}_{S}=\frac{1}{j}\sum_{i=1}^{j}\left(M_{i}\right)$$
  A larger value of Average AS means a higher privacy level.
2. PLA.
  As we mentioned above, we propose to measure PLA in different cases based on the sensitivity of the node’s area. The first case is when the source node enters a non-sensitive area. The second case is when the source node enters a sensitive area where it is important to further decrease the probability of the attacker to successfully link the source node with its new DID. The mathematical models for both cases are as follows:
  - PLA for non-sensitive area *(${\mathrm{PLA}}_{\mathrm{NS}}$*):
    Here, we derive an expression for ${\mathrm{PLA}}_{\mathrm{NS}}$. Since the source node along with the participating nodes ${\mathrm{NPN}}_{i}$ for change ***i*** will synchronously change their DID during the silence period, there will be ${\mathrm{NPN}}_{i}$ + **1** new DIDs. Therefore, the probability that the attacker will succeed in linking the old DID of the source node with its new DID after the silence period for a non-sensitive area for change ***i*** is given by Equation (6).
    (6) $${\mathrm{PLA}}_{i_{\mathrm{NS}}}=\frac{1}{{\mathrm{NPN}}_{i}+1}$$
    where 1 refers to the source node.
  - The Average PLA for the non-sensitive area (Average ${\mathrm{PLA}}_{\mathrm{NS}}$) per change is given by Equation (7) below:
    (7) $$\mathrm{Average}\;{\mathrm{PLA}}_{\mathrm{NS}}=\frac{1}{j}\sum_{i=1}^{j}\frac{1}{{\mathrm{NPN}}_{i}+1}$$
  - PLA for sensitive area (${\mathrm{PLA}}_{S}$):
    For the sensitive area case, since all immediate neighboring nodes ($M_{i}$) of the source node are forced to participate and enter a silence period, so
    $${\mathrm{NPN}}_{i}=M_{i}$$
    Thus, the PLA for the sensitive area (${\mathrm{PLA}}_{S}$) for change ***i*** is given by Equation (8) below:
    (8) $${\mathrm{PLA}}_{i_{S}}=\frac{1}{M_{i}+1}$$
  - The Average PLA for the sensitive area (Average ${\mathrm{PLA}}_{S}$) per change is given by Equation (9) below:
    (9) $$\mathrm{Average}\;{\mathrm{PLA}}_{S}=\frac{1}{j}\sum_{i=1}^{j}\frac{1}{M_{i}+1}$$
  A smaller value of PLA means a higher privacy level.

### 4.3. Security Analysis

We evaluate the proposed scheme’s security strength in terms of its ability to resist potential security attacks as below:

- Linkability Attack The linkability attack uses the transmitted information to link the dummy ID with the target node. To resist this kind of attack, our method relies on the use of a silence period to prevent linkability and tracking during the identifier-changing process. All the participating nodes and the source node enter the silence period synchronously. Since the DIDs of the participating nodes and the source node after the silence period are different from the ones before the silence period, the attacker will be confused and its chance for successful tracking of the source node (target) will be reduced.
  In addition, our proposed sensitive area feature further restricts linkability attacks to link the target node with its dummy ID beyond existing schemes as it forces all the immediate nodes to participate.
- Eavesdropping Attack
  In the eavesdropping attack, the attacker listens to the communication between the nodes in the network to obtain the desired information. To resist this kind of attack, our method uses a pool of temporary DIDs for each node for communication purposes. Additionally, a pseudonym location is used instead of the real one to protect the real location. Therefore, the attacker will not be able to obtain any useful information about the node’s real ID and its location.
  In addition, our proposed sensitive area feature further restricts eavesdropping attacks to link the target node with its dummy ID beyond existing schemes as it forces all the immediate nodes to participate.

### 4.4. Simulation Analysis

To test our model, a MATLAB simulation was developed for the proposed scheme by using the parameters in Table 3.

#### Simulation Environment and Entities

In the simulation, we divided the network into grid cells, and we assigned a unique identifier number to each grid cell starting from n = 2 to n + 1 to avoid the case when n = 0 or n = 1. Once a simulation starts, the gateways will be created and randomly distributed in the grid within the first two seconds. Then, the nodes will be created and randomly distributed in the grid within five seconds. More details about the simulation entities are included below:

- Grid:
  The grid class has several properties, including grid cell size, grid cell length, gateway objects, node objects, and an interrupt list. The grid cell size simply refers to the grid cell length and width in terms of pixels, where each pixel represents a certain region in the actual physical grid cell. The gateway and node objects are other class instances affiliated with the grid simulation. The interrupt list is a list of time interrupts that refer to all future expected events at which the simulation pauses, monitors occurring events, and updates the status of the grid cell accordingly. The class has a constructor that initializes all parameters of the grid at the start of the simulation according to user preferences. The class uses an update method that is called when a time interrupt occurs. This method updates the status of the grid, including the states of the nodes and gateways contained within the grid.
- Gateways:
  The gateway class also has distinct properties, including ID, position, velocity, registration tables, and associated node DIDs. The ID is a distinct code used to distinguish between different gateways. The position and velocity are kinematic measures for the motion of the actual gateway and are both measured using the international system of units SI (meters, meters/second, etc.). Each gateway has several nodes it is supposed to serve, where each of these nodes has a pool of DIDs that are also registered in each gateway’s memory. The gateway also has a constructor that initializes the values of all of these properties prior to the simulation run. The gateway has a set of methods it uses to achieve its intended goals, including the registration service function, which serves nodes that issue a registration request. This method initializes the registration table of the node, which includes data such as the node ID, associated gateway ID, gateway grid cell number, and registration timestamp. The gateway also has another method that serves DID change requests. The gateway first checks if the message is infected by comparing the node’s confirmation message’s registration table against the registration table recorded in its memory. If the registration tables match, the gateway will check the number of voting nodes. If most associated nodes’ DIDs are close to be expired, the gateway approves the request (APP = 1). Otherwise, the gateway does not approve the request, and the node obtains a new expiration deadline. The gateway also has a kinematic state update method that updates the gateway’s kinematic position and velocity.
- Nodes: The node object has several properties, including ID, DID lifetime, time of creation, position velocity, turn-off flag, and expiration flag. The DID lifetime is set to sixty seconds from the moment of creation. The time of creation itself is the time at which the node shows up in the simulation, which is randomly set for each run within the first five seconds of run time. The turn-off flag is raised to true when the node enters a sensitive region; otherwise, it is set to false. The expiration flag indicates that the node’s DID is close to expire and needs to be changed. The node methods include an update method, which updates the kinematic states of the node, as well as node flags.

### 4.5. Simulation Results

**Figure 8:**
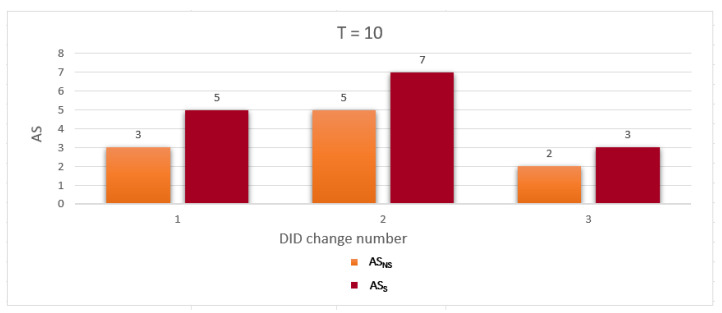
Anonymity of the source node (AS) for each DID change with 10 nodes.

**Figure 9:**
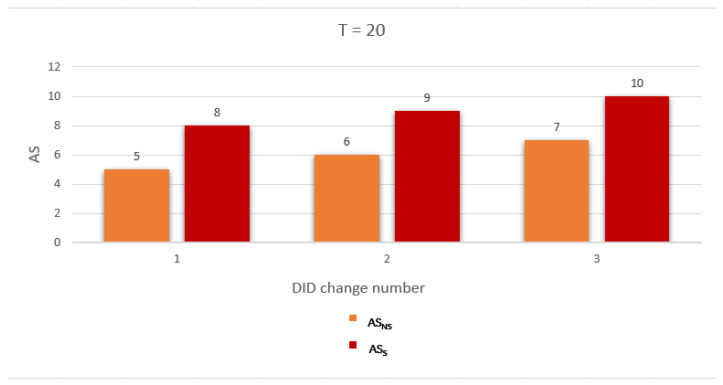
Anonymity of the source node (AS) for each DID change with 20 nodes.

**Figure 10:**
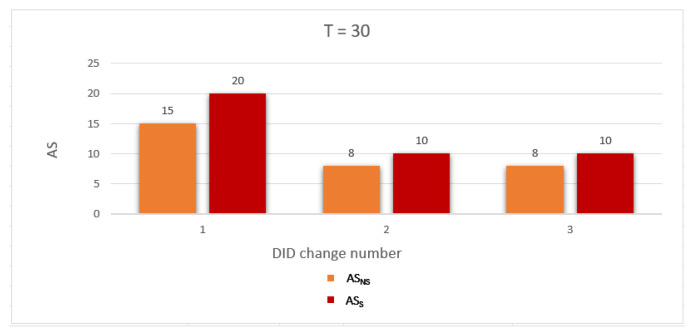
Anonymity of the source node (AS) for each DID change with 30 nodes.

**Figure 11:**
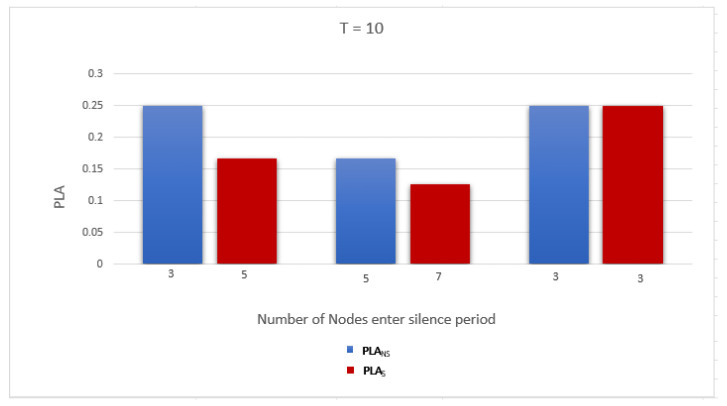
Probability of linkability (PLA) of the source node in a network with 10 nodes.

**Figure 12:**
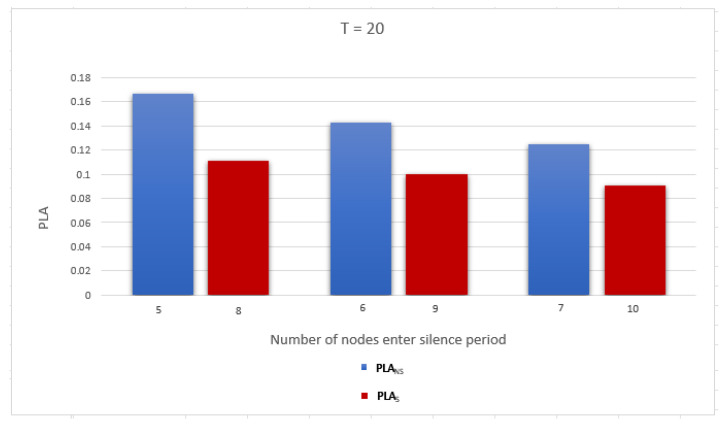
Probability of linkability (PLA) of the source node in a network with 20 nodes.

**Figure 13:**
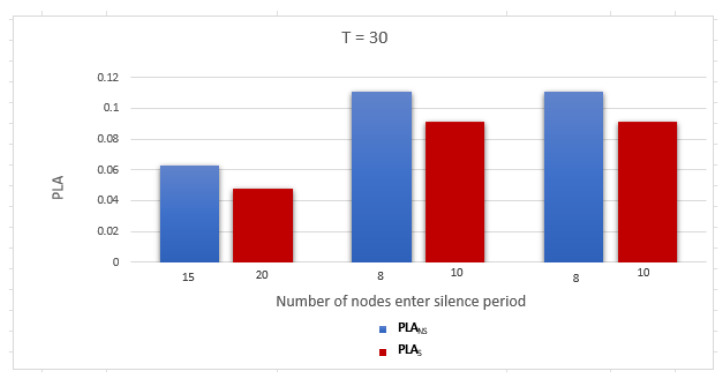
Probability of linkability (PLA) of the source node in a network with 30 nodes.

To evaluate the performance of the proposed work, we use two metrics related to measuring the privacy level of the location information and identity as follows: Average anonymity of the source node (Average AS) and Average probability of linkability (Average PLA) per DID change. The simulation was run for a network with two gateways and number of nodes = 10, 20, and 30. The simulation results are discussed below:

- Average anonymity of the source node per DID change (Average AS): In our simulation, to measure the further enhancement introduced by applying the sensitive area concept, we measured Average AS for the source node per DID change in two cases: when a sensitive area concept was applied, ${\mathrm{AS}}_{S}$, and when a sensitive area concept was not applied, ${\mathrm{AS}}_{\mathrm{NS}}$. Figure 8, Figure 9 and Figure 10 show ${\mathrm{AS}}_{\mathrm{NS}}$ and ${\mathrm{AS}}_{S}$ results with 10, 20, and 30 nodes in the network, respectively.
  For ${\mathrm{AS}}_{\mathrm{NS}}$ results, since AS equals the number of NPN as we mentioned above, more participating nodes will increase the source node anonymity. For ${\mathrm{AS}}_{S}$ results, all the immediate neighboring nodes are forced to participate and change their DIDs whether their DIDs lifetime is about to expire or not. Thus, the number of nodes entering the silence period will be equal to or larger than the number of nodes entering the silence period in the ${\mathrm{AS}}_{\mathrm{NS}}$ case, which means AS for the source node is further enhanced.
  Table 4 shows the Average $AS_{NS}$ and Average $AS_{S}$ per DID change. It is clear that applying the sensitive area concept further enhances the average anonymity of the source node for all different numbers of nodes in the network.
- PLA Figure 11, Figure 12 and Figure 13 present the PLA results for the total number of nodes T = 10, 20, and 30. It can be seen that in the ${\mathrm{PLA}}_{\mathrm{NS}}$ case, increasing the number of nodes entering the silence period decreases the value of the corresponding ${\mathrm{PLA}}_{\mathrm{NS}}$. In addition, further enhancement is achieved in the ${\mathrm{PLA}}_{S}$ case, since all immediate neighbors (M) of the source node are forced to enter the silence period and change their DIDs.
  Table 5 shows the Average ${\mathrm{PLA}}_{\mathrm{NS}}$ and Average ${\mathrm{PLA}}_{S}$ per DID change. It is clear that applying the sensitive area concept further decreases the Average PLA of the source node for all different numbers of nodes in the network.

To the best of our knowledge, our work is the only work that focuses on the IoBT environment, which has different characteristics and strong security requirements. Moreover, the security of the node location once entering a sensitive area is critical. Thus, we introduced the sensitive area concept. To investigate its impact, we evaluated the proposed scheme with and without it. Our results presented above showed that having this feature further enhances the Average AS and PLA per DID change.

## 5. Conclusions

IoBT networks are very different from conventional IoT networks because of challenges specific to the battlefield, such as lack of infrastructure, the heterogeneity of equipment, and attacks. Real-time location data collection is essential for battle efficiency in war scenarios and is dependent on network connectivity and information exchange when an adversary is present. The mission depends on exchanging and sending location information to achieve connectivity and ensure the security of soldiers and equipment. Transmissions will include information about soldier/equipment locations, identities, and trajectories. If a malicious attacker obtains this information, then it reconstructs the whole trajectory of a target node and monitors its movements.

The focus of this paper was to secure location and identity information in IoBT networks. To protect the source node identity and to minimize linkability and tracking, dummy IDs and silence period concepts are used in our scheme. In addition, it is more critical to further enhance the anonymity of the source in sensitive areas of the battlefield. Thus, we proposed the sensitive area location privacy enhancement concept. In this case, when the source node enters a sensitive area the gateway forces all the immediate neighboring nodes to participate by entering the silence period to change their DIDs.

Moreover, to protect the location information, an additional security layer is proposed to create a pseudonym location for a source node.

A Matlab simulation was developed to evaluate our scheme in terms of average anonymity and average probability of linkability of the source node. The results obtained demonstrated the utility of the concepts used in our scheme in enhancing the security of location and identity information. In addition, they showed the significance of applying the sensitive area concept in IoBT networks as it enhances the anonymity and decreases the linkability of the source node.

## Figures and Tables

**Table 1:** The comparison of our work to recent deception-based related works.

Ref/Year	Changing ID Information	Changing Location Information	Environment/ Applications	Sensitive Areas Location Privacy Enhancement	Silence Period
[16]/2022	√	×	Internet of vehicles (IoV)	×	√
[28]/2022	×	√	LBS	×	×
[39]/2022	×	√	Mobile Crowdsensing (MCS)	×	×
[42]/2022	√	×	IoV	×	√
[18]/2021	√	×	IoV	×	√
Our work	√	√	IoBT	√	√

**Table 2:** Notations of the proposed work.

Notation	Meaning
DID	Dummy ID
N	Source node
G	Gateway
APP	Approval status could be 0 or 1
K	The Threshold.
n	Unique integer identifier number for the grid cell
L	The location longitude of the node
$\bar{L}$	The pseudonym location longitude of the node
D	The location latitude of the node
$\bar{D}$	The pseudonym location latitude of the node
E	The DID status could be 1 or 0
U	Urgent status
M	The number of immediate Neighboring nodes
T	The total number of the nodes in the network

**Table 3:** The parameters.

The Parameter	The Values
DID lifetime	60 s
Node communication range	500 m
Total number of nodes (T)	10, 20, 30
Number of gateways	2
Number of sensitive regions	500
Grid cell length	100
simulation time	200 s

**Table 4:** Average anonymity (Average AS) of the source node per DID change.

Total Number of Nodes (T)	Average ${\mathrm{AS}}_{\mathrm{NS}}$	Average ${\mathrm{AS}}_{S}$
10	3.7	5
20	6	9
30	10.3	13.3

**Table 5:** Average probability of linkability (PLA) of the source node.

Total Number of Nodes (T)	Average ${\mathrm{PLA}}_{\mathrm{NS}}$	Average ${\mathrm{PLA}}_{S}$
10	0.22	0.18
20	0.095	0.076
30	0.14	0.1

## Data Availability

Not applicable.
